# Comparing adherence to MDR-TB treatment among patients on self-administered therapy and those on directly observed therapy: non-inferiority randomized controlled trial

**DOI:** 10.1186/s13063-023-07314-z

**Published:** 2023-05-12

**Authors:** Clara Wekesa, Christine Sekaggya-Wiltshire, Stella Zawedde Muyanja, Ivan Lume, Maria Sarah Nabaggala, Rosalind Parkes-Ratanshi, Susan Adakun Akello

**Affiliations:** 1 Infectious Diseases Institute, Makerere University, Kampala, Uganda; 2 Department of Public Health, Cambridge University, Cambridge, UK; 3 TB unit, Mulago National Referral Hospital, Kampala, Uganda

**Keywords:** Multi-drug-resistant tuberculosis, Medication events monitoring system technology, Directly observed therapy, Differentiated care, TB treatment adherence, TB treatment outcomes, Resource-limited settings, Sub-Saharan Africa, TB drug PK levels

## Abstract

**Background:**

Adherence is key to the treatment success of multi-drug resistant tuberculosis (MDR-TB) and prevention of community transmission. Directly observed therapy (DOT) is the recommended approach for the management of patients with MDR-TB. Uganda implements a health facility-based DOT approach where all patients diagnosed with MDR-TB report to the nearest private or public health facility for daily observation of ingesting their medicines by a health care provider. Directly observed therapy is very costly for both the patient and health care system. It follows the assumption that MDR TB patients have a history of poor adherence to TB treatment. But only 21% of MDR-TB patients notified globally and 1.4–12% notified in Uganda had been previously treated for TB. The shift to all oral treatment regimen for MDR-TB provides an opportunity for the exploration of self-administered therapy for this group of patients even with use of remotely operated adherence technology. We are conducting a non-inferiority open-label randomized controlled trial to compare adherence to MDR-TB treatment among patients on self-administered therapy (measured by Medication Events Monitoring System (MEMS) technology) with a control group on DOT.

**Methods:**

We plan to enrol 164 newly diagnosed MDR-TB patients aged ≥ 8 years from three regional hospitals based in rural and urban Uganda. Patients with conditions that affect their dexterity and ability to operate the MEMS-operated medicine equipment will not be eligible to participate in the trial. Patients are randomized to either of the two study arms: self-administered therapy with adherence being monitored using MEMS technology (intervention arm) or health facility-based DOT (control arm) and will be followed up monthly. Adherence is measured by the number of days the medicine bottle is open to access medication as recorded by the MEMS software in the intervention arm and treatment complaint days as recorded in the TB treatment card in the control arm. The primary outcome is the comparison of adherence rates between the two study arms.

**Discussion:**

The evaluation of self-administered therapy for patients with MDR-TB is important to inform cost-effective management strategies for these patients. The approval of all oral regimens for the treatment of MDR-TB provides an opportunity for innovations such as MEMS technology to support sustainable options for MDR-TB treatment adherence support in low-resource settings.

**Trial registration:**

Pan African Clinical Trials Registry, Cochrane #PACTR202205876377808. Retrospectively registered on 13 May 2022

**Supplementary Information:**

The online version contains supplementary material available at 10.1186/s13063-023-07314-z.

## Administrative information

**Table:** 

**Title**	Comparing adherence to MDR-TB treatment among patients on self-administered therapy and those on Directly Observed Therapy: Non Inferiority Randomized Controlled Trial (**WISECAP Study**)
**Trial registration**	Pan African Clinical Trials Registry, Cochrane #PACTR202205876377808 on 13^th^ May 2022
**Protocol version**	23.09.21 Version 1.2
**Name and contact information for the trial sponsor**	This Trial is under funding by Janssen Global Public Health, a division of Janssen Pharmaceutica NV, under grant number 1550786 Turnhoutseweg 30, B-2340 Beerse, Belgium
**Names, affiliations, and roles of protocol contributors**	Clara Wekesa^1^, Christine Sekaggya Wiltshire^1^, Stella Zawedde Muyanja^1^, Ivan Lume ^1^, Maria Sarah Nabagalla^1^, Rosalind Parkes-Ratanshi^1,2^, Susan Adakun Akello^3^ 1. Infectious Diseases Institute, Makerere University 2. Department of Public Health, Cambridge University 3. TB unit, Mulago National Referral Hospital SA, CS, SZ, RPR and CW conceived the study, participated in the proposal and protocol development. SN wrote up the statistical methods of the study and IL contributed to development of the study tools. All authors read and approved the final manuscript.
**Role of sponsor**	The funding body did not take part in the conception of the experiment and will be independent of the study conduct, analysis and interpretation of the results. The funding body did not participate in the write-up of this manuscript.
**Roles and responsibilities: committees**	The Data Monitoring Board and the Trial Steering Committee will make recommendations concerning the conduct of the trial based on periodically shared data progress reports and interim results. The independent Trial Monitor will make recommendations of the adherence to study protocol and operating procedures as well as ensuring participant safety during the course the study.

### Introduction

#### Background and rationale

Multi-drug-resistant tuberculosis (MDR-TB), which is resistance to both isoniazid and rifampicin, poses a challenge to TB control programmes globally because of the risk of worse treatment outcomes including death compared to drug-sensitive TB [1]. In 2020, 71% (2.1 of 3 million) of patients found to have pulmonary TB tested positive for rifampicin resistance, a ten percent increment from the previous year [2]. Treatment success for drug-resistant TB remains sub-optimal, below the World Health Organization (WHO) target of 90%, increasing risk for disease relapse and continued community transmission. In Uganda, for the period between July 2019 and June 2020, 513 patients tested positive for rifampicin resistance and or MDR-TB (RR/MDR-TB) and the treatment success rate in the same period was 77.9% [3]. Global mortality resulting from MDR-TB remains high, and more so among people co-infected with HIV/AIDS. Mortality rate among people living with HIV (PLHIV) is 20% in comparison to 15% among mono-infected MDR-TB patients on treatment [4]. Observational studies done in Uganda have reported a similar mortality rate of 16–18% among MDR-TB patients on treatment [4, 5].

In an effort to counter the effects of non-adherence, including poor treatment outcomes and transmission of resistance strains, directly observed therapy (DOT) was adopted as a strategy, where patients are observed as they take their daily dose by either a community health care worker, family member or health care worker. The observation may occur at a health facility when the patient reports for their daily dose or may entail the health provider visiting the patient at their home premise. Uganda implements a health facility-based DOT approach where all patients diagnosed with MDR-TB report to the nearest private or public health facility for DOT. Although treatment success rates on patients treated using the DOT strategy in Uganda have gradually improved from 70.6% in 2018 to 77.9% in 2020, these rates are still below the 90% target set by the WHO. Factors that contribute to low treatment success rates with DOT include lack of financial resources to make daily clinic visits, poor road infrastructure, long distances to nearest health facility, insufficient and overworked health care workers, and overburdened facilities [6, 7]. Therefore, innovations that can support remote monitoring of MDR-TB treatment should be explored to reduce time and financial costs. A systematic review on strategies to improve adherence to MDR-TB treatment demonstrated that community DOT had varied effectiveness and that barriers to achieving adherence are patient-based; there is therefore a need to develop personalized interventions [8].

The Medication Events Monitoring System (MEMS) (AARDEX®) is a portable smart medication device designed to monitor treatment adherence by transmitting electronic information on the number of times the medicine cap is opened, specifying dates and times. This information is taken to correlate with actual times that patients ingest their medication and can be used to validate the accuracy of self-reported adherence. The device is operated by a long-lasting battery and is able to transmit software information in real time and store information for many months following battery outage. The information on patient non-adherence as captured by MEMS technology has been shown to accurately correlate with treatment outcomes among people living with HIV [9]. In addition, the device has also shown to outperform self-report or pill counts and is acceptable to patients who found it easy to open and carry it on their person [9, 10].

We plan to conduct an open-label randomized non-inferiority clinical trial among patients receiving MDR-TB treatment in Uganda to compare adherence rates among those using self-administered therapy with MEMS technology vs DOT, which is the current standard of care in Uganda for MDR-TB. We will correlate adherence information with serum drug concentrations and compare determined treatment outcomes between the two groups.

## Explanation for choice of comparators

For the purposes of this study, we shall be using patients on standard of care (DOT), as the comparator group. This comprises patients with MDR-TB who have to report daily to a DOTS facility to be observed as they ingest their medication. DOT is the standard of care for MDR-TB patients and presently the only alternative for patient follow-up.

## Specific objectives

The primary objective of the study is to determine if adherence to MDR-TB treatment among patients on self-administered therapy (measured by MEMS technology) is non-inferior to that among patients on directly observed therapy (DOT). The secondary objectives of the study include determining the correlation between MDR-TB drug serum concentrations and adherence as measured by MEMS technology and comparing treatment outcomes between MDR-TB patients on self-administered therapy and standard DOT

## Trial design

This study will be conducted as a multi-centre open-label randomized clinical trial with two parallel arms and 1:1 enrolment ratio (82 patients in the intervention arm and 82 controls). We shall enrol a total of 164 participants newly diagnosed with pulmonary MDR-TB or rifampicin-resistant TB (RR-TB) from 3 TB tertiary care centres from 2 regions of the country.

### Methods

We used the SPIRIT reporting guidelines in reporting our study methods [11].

#### Study setting

This study will be conducted in Mulago National Referral Hospital in the central region and Lira regional referral hospital in the northern region. These hospitals register the highest number of MDR-TB patients in Uganda. Both these hospitals are public government hospitals not for profit. The hospitals house specialized TB wards and clinics, operating a weekly TB clinic to follow up patients with drug-sensitive and drug-resistant TB on therapy and to initiate therapy for newly diagnosed patients. Being referral hospitals, these facilities receive patients referred from health facilities within a radius of up to 30km and in some cases even further.

#### Diagnosis and management of MDR-TB

Diagnosis of MDR-TB is based on Gene Xpert® testing of sputum for identification of resistance to rifampicin (RR-TB) followed by culture and drug susceptibility testing (DST) for phenotypic characterization; however, treatment is started prior to the receipt of culture and sensitivity results due to long turnaround time of 10–16 weeks. Treatment options for MDR-TB/RR-TB currently include:

- Category 1—A bedaquiline-based injection-shorter treatment regimen (STR) (9 to 12 months) reserved for MDR-TB/RR-TB patients who have not been previously treated with second-line drugs and in whom resistance to fluoroquinolones and second-line injectable agents has been excluded. Drug options for shorter treatment regimen include an intensive phase of 4 months (extended to 6 months in case of delayed sputum smear conversion) containing high-dose gatifloxacin or moxifloxacin, kanamycin, prothionamide, clofazimine, high-dose isoniazid, pyrazinamide, and ethambutol followed by a continuation phase of 5 months containing gatifloxacin or moxifloxacin, clofazimine, ethambutol and pyrazinamide [12].
- Category 2—The longer treatment regimens (18 to 20 months) based on core backbone agents but individualized depending on age, fluoroquinolone resistance, and site of disease.

According to standard of care, patients with MDR-TB are managed as in-patients at central MDR treatment facilities (which include Mulago and Lira hospitals) in the intensive phase of treatment after which they are discharged and required to report to the central MDR treatment facility monthly for review and drug refills. Following discharge, during the continuation phase, patients are required to report daily to the nearest private or public facility registered under DOT programme [13]. Monthly reviews during routine care include safety laboratory investigations including a full blood count, liver and renal function tests, sputum culture, DST and adherence counseling.

#### Eligibility criteria

We shall enrol consented patients with newly diagnosed pulmonary MDR-TB and RR-TB patients aged ≥8 years, initiated on oral MDR-TB therapy (category 1 or 2). Patients with any condition that inhibits the use of the MEMS device, e.g. dexterity or debilitating arthritis, will be excluded from participation.

#### Intervention arm

For this trial, we shall use two kinds of MEMS-operated devices, the MEMS cap and the MEMS Electronic DosePak® (EDP). At enrolment, patients on the self-administering arm will be provided with both devices; the MEMS cap will be placed over a medicine bottle containing bedaquiline. The concomitant medication will be packaged in a convenient pack containing all tablets to be swallowed each day and placed into the medication box with the EDP® technology Patients will be instructed on how to use the MEMS devices. Each time a patient opens up the cap over the bedaquiline bottle or the lid of the medicine box, the time and date of this event will be immediately captured. Adherence data from the MEMS devices will be downloaded by the study team every month during the routine visits. The devices will provide information on specific calendar dates and times when the devices were opened. Information on the calendar days with event markings to signify the date and time when medication containers were opened will be displayed on a computer. The software will contain two separate input calendars for both the MEMS cap and the EDP box for each participant. This same adherence data will be shared with primary clinician and used to tailor adherence counselling given to patients, exploring patterns of missed medication and brainstorming for solutions with the patient (Table 1).

**Table 1:** Description of study arms

**Control: DOT**	**Intervention: self-administered therapy with MEMS technology**
Patients report daily to the nearest health unit to be observed by a health care provider as they ingest their medication Behavioural: Adherence counselling. Patients will receive adherence counselling as scheduled by their primary care health providers	Device: MEMS CAP without LCD display and EDP® technology on medicine box. Standard MEMS devices record the time and date when the bottle is opened The MEMS technology automatically complies drug dosing history information by electronically recording the date and time of each opening of the medication cap and or box Behavioural: Adherence counselling. Patients will receive adherence counselling as scheduled by their primary care health providers

## Interventions: modifications

Participants may withdraw from the study at any time at their own request, or they may be withdrawn at any time at the discretion of the investigator or sponsor for safety or behavioural reasons, or the inability of the subject to comply with the protocol required schedule of study visits or procedures. Patients who are withdrawn at any time of the study may be replaced.

### Adherence to interventions

Participants on the intervention arm will be given monthly study visits in which the adherence software shall be read to determine information on use of the medicine cap and box. Based on the information displayed, study personnel provide tailored adherence counselling to the participants. For participants below the age of 18 and under guardianship, their caretakers will be included in the adherence counseling sessions.

### Determination of TB treatment outcomes

At the end of therapy treatment (9 months for participants that qualify for shorter MDR-TB/RR-TB treatment regimen and 18 months for those selected for the longer MDR-TB/RR-TB treatment regimen), clinical and laboratory information is routinely compiled by each MDR-TB centre and forwarded to the National TB Program (NTP) review committee for discussion on consensus on treatment outcome based on a specified criterion (Table 2). TB treatment outcomes for this study will be as determined by the review committee.

**Table 2:** Description of MDR-TB treatment outcomes

**Outcome**	**Definition**
Time to culture conversion	Time from initiation of treatment to the time point where the patient has two consecutive negative cultures
Cured	1. A patient who has culture converted and 2. Received treatment for a total duration of 9–11 months and 3. Has at least 3 consecutive negative TB cultures at least 30 days apart and 4. No evidence of clinical deterioration
Treatment completed (success)	1. A patient who has culture converted and 2. Received treatment for a total duration of 9–11 months and 3. Has less than 3 consecutive negative TB cultures at least 30 days apart and 4. No evidence of clinical deterioration
Loss to follow-up	A patient with treatment interrupted for 2 consecutive months and more
Treatment failure	1. A patient who failed to culture convert by month 5 (shorter regimen) or month 6 (longer regimen) or 2. In the initial 6 months of treatment more than 2 of 5 cultures are positive or 3. Treatment stopped due to adverse events 4. Permanent discontinuation of two Group A drugs (BDQ, LZD, or LFX) in the first 6 months of the modified shorter regimen or 5. More than 2 new drugs added because of poor clinical response or 6. Panel decision to terminate any further DR-TB treatment
Died	A patient who died for any reason during treatment

#### Study outcomes

Our primary outcome is the adherence rates among MDR-TB patients on self-administered therapy vs DOT. Our secondary outcomes include (1) drug concentrations and adherence data for patients on self-administered therapy vs DOT; (2) proportion of patients with sputum conversion at 2, 8 and 11 months in each study arm; and (3) MDR-TB treatment outcomes (cure, treatment completion, loss to follow-up or death) by study arm.

### Sample size

Based on records from the NTP, treatment success rate for MDR-TB is 77.9%; from clinical trials, adequate adherence has been defined as 76–80% adherence rate; and from a meta-analysis, it has shown that 70% of patients on MDR-TB therapy are adherent [14, 15]. Our sample size calculation assumed that the adherence rate for the patients using MEMS technology is ≥ 70%, with study power of 0.9, precision of 0.05, and 20% loss to follow-up rate. We therefore require a sample size of 164 patients (82 in each arm) to demonstrate a 19% increment in MDR-TB treatment success. The intervention (MEMS technology) approach is considered non-inferior (19%) to the control (DOT) approach if the lower limit of the 95%CI of the difference between the two groups (i.e. MEMS group minus DOT group) is > −10%.

### Recruitment

In order to enrol the required sample size in the stipulated time frame of the study, we shall enrol from sites with high volume numbers of MDR-TB in the country, which include Mulago National Referral Hospital and Lira Regional Referral Hospital. We shall also endeavour to enrol all eligible participants at each clinic day at both sites.

#### Randomization and allocation of intervention

Blinded randomization will be performed by the study team (medical officer or study nurse) at the time of enrolment using an automated randomization sequence within the Redcap data capture tool. The randomization will be done in real time on the Redcap tool as it will be Internet enabled. Participants will be randomized to either intervention arm (arm 1; self-administered therapy) or control arm (arm 2; DOT). Stratified randomization will be performed according to age group and gender. Due to the nature of the intervention and organization of patient care, it will not be possible to blind either participants or study staff to the study arm allocation

#### Control arm

Patients randomized to the control arm will receive daily DOT provided by a peripheral health facility close to their residence. Under the DOT programme, patients report to the nearest DOT provider facility daily and drug intake is observed. On each visit, the TB card is ticked off in correspondence with the number of days they made a visit to the health centre and this card is retained at the health facility. At the monthly visits, the TB card is taken to the central MDR treatment facility for review by the managing clinician. At this point, the study team will record the number of doses taken/missed according to the TB card. Participants will receive adherence counselling on all routine visits at the central facility (Table 1).

### Allocation concealment mechanism

The designing of the randomization sequence within the Redcap tool was performed by a biostatistician who is blinded from the participant enrolment activities. The variables used to design the randomization sequence included gender and age group. At the stage of enrolment, the randomization is conducted in real time on the Internet-enabled Redcap tool by the member of the study team conducting the enrolment. The study team have no control over the randomization sequence.

### Allocation implementation

The study team (medical officer and or study nurse) having determined participant eligibility enter the participant gender and age into the automated randomization sequence within the Redcap tool and click a button to determine study allocation and or study arm in real time.

### Blinding (masking)

Given the nature of the intervention (a visible medicine box and the requirement not to report to a DOT facility daily), it was not feasible to blind study participants, primary care providers, the study team and or the data manager.

### Data collection plan

All participants will be reviewed by the study team monthly during routine visits (Table 3). On the initial visit, participants will be taken through a screening questionnaire to assess eligibility. Patients found to be eligible will then be taken through the informed consent process in the language of their preference. Enrolled participants will provide demographic information including age and gender. At each monthly study visit, participants will be taken through an interviewer-led questionnaire to capture clinical history (reported adverse events, treatment outcomes) and adherence information (adherence data downloads from MEMS devices, adherence data from TB treatment cards, pills counts, and participant-reported adherence on days of treatment missed). Adherence data will be used to provide tailored counselling for each participant to encourage treatment completion and this data will be shared with the participant’s primary clinician.

**Table 3:**
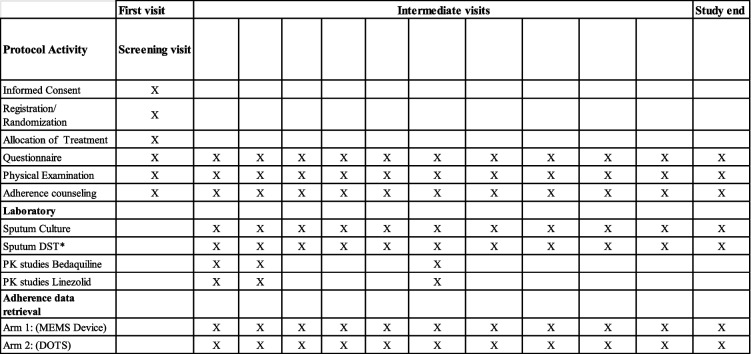
Description of study procedures

^*^Drug susceptibility testing: sputum culture and sputum DST will be provided as standard of care by the health facility

## Laboratory assessments

Blood sampling to measure the concentrations of bedaquiline and linezolid will be conducted on months 2, 8 and 11 of treatment. Drug intake on the days for blood sampling will be performed by DOT and blood samples taken off prior to drug intake and 1-h post-drug intake. The blood samples will be placed in cooler boxes and transported to the laboratory on dry ice for samples from Lira Hospital and in a cooler box for samples from Mulago Hospital, processed and stored at −4 to −80 °C. Pharmacokinetic analysis will be performed at the Infectious Diseases Institution (IDI) translation laboratory. Quantification of the drug concentrations will be performed in batches using validated high-performance liquid chromatography (HPLC) and mass spectrometry at the Translation Laboratory of the Infectious Diseases Institute, Makerere University using MS.

Sputum smears and cultures will be performed as part of routine care and testing conducted at the government reference laboratory.

The study follow-up duration will be 24 months to cater for patients on both short- and long-term oral MDR-TB therapy. Treatment duration will be approximately 9 to 18 months: 9 months for participants that qualify for shorter MDR-TB/RR-TB treatment regimen and 18 months for those selected for the longer MDR-TB/RR-TB treatment regimen.

To ensure data quality, study information is captured with an e-questionnaire in Redcap that has been designed to send real-time notifications for inputted variables that may be out of range. The tool also does not accept for the person collecting data (medical officer and or study nurse) to verify the tool, but is redirected to the study coordinator and Data Manager to do the final verification for correct and missing data. In addition, the entire study team was trained afore on the use of the study Redcap tool and on other study-related standard operating procedure manuals including the collection of blood specimens for continued reference. In the event of a protocol violation, retraining on study protocol and procedures will be conducted.

### Retention of study participants in the Trial

For all persons approached to participate in the study, full information will be provided about the trial its aims, study procedures and duration. Participants will be given an opportunity to ask any questions they may have at the time of consent and any other time of the study. Data from participants that have withdrawn and or been withdrawn from the study will be retained for sub-analysis. All participants that have been withdrawn from study participation will be replaced to ensure the sample size is achieved.

### Data management

Clinical data will be entered into a study-specific database by trained designated study staff on a regular basis from completed Case Record Forms (CRF) using Redcap. Information will primarily be captured using Redcap and CRFs will be printed out for filing and storage. Case Record Forms and other source documents will be kept in locked cabinets. No participant-identifying information will be disclosed in any publication or at any conference activities arising from the study. The participant data captured in the Redcap tool will be accessed in real time by the data manager, who will provide data queries for the study team to address. In addition, the Redcap data collection tool has been fashioned to generate automated checks on entry of certain variables that are out of range. Data management procedures are detailed in the standard operating manual.

### Statistical: outcomes

#### Adherence assessment

The primary outcome is the overall adherence rates among MDR-TB patients on self-administered therapy and on DOT. For the participants on the intervention arm, opening of the devices will be equated to a patient swallowing their medication and adherence will be derived from the number of days the device was opened as a proportion of the total number of days in the entire period of their MDR-TB treatment. Patients on the intervention arm will be required to take no less than 70% of their medication to be considered as having good adherence. For participants on the control arm (DOT), adherence information will be obtained from their TB treatment cards. Adherence rates will be calculated from the number of days that they report to the DOTS facility as a proportion of the total number of treatment complaint days in the entire period of their MDR-TB treatment. Participants who take their medication at least 80% of the time (missing no more than 5 days each month) will be considered as having good adherence.

The primary analysis will be a comparison of overall adherence in each arm. Adherence by self-administration (proportion of expected openings of the MEMS devices) and DOT (proportion of DOT visits) will also be compared at 2 months and 6 months in the intervention and control arms. The chi-square test and 1-way analysis of variance models will be used to compare outcomes by randomization arm. We shall use independent samples *t*-test to test for statistical difference mean adherence rates in the patient in the intervention and control arms. We will use an intent-to-treat approach (analysed all participants regardless of drug refills) to compare adherence outcomes by randomization arm using a generalized estimating equations model using repeated measures with identity link, exchangeable correlation structure and robust variance estimates.

#### Secondary outcomes

To assess correlation of specific MDR-TB drug serum concentrations with observed adherence, bedaquiline and linezolid pre-dose concentrations (C_trough_) will be used. To cater for those who may be adherent but have low bedaquiline or linezolid concentrations, we will use of cut-off value of bedaquiline C_trough_ from previous studies, below which a participant will be classified as non-adherence. Linezolid will be used to assess more recent adherence than bedaquiline.

We will compare time to sputum culture conversion in the intervention and control arm using time to event analysis. We shall also use chi-square testing to compare treatment success rates in the intervention and control arms. Treatment success will be evaluated using a composite outcome that includes TB cure (for microbiologically diagnosed patients) and TB treatment completion (for clinically diagnosed TB). We will undertake per-protocol and intention to treat analyses to compare TB cure rates in the two study arms. We will determine the proportions of patients retained in care of retention in care at 2 and 6 months in the two study arms using chi-square tests. Study outcomes by randomization arm will be compared using a generalized estimating equations (GEE) model using repeated measures with identity link, exchangeable correlation structure and robust variance estimates. Adjusted coefficients along with their corresponding 95% CIs will be generated. Statistical significance will be set at the *P* < .05 level and statistical analyses performed using Stata version 14 (StataCorp, College Station, TX, USA).

#### Additional analyses and handling of missing data

Interim data analysis will be conducted once 50% of the overall sample size has been achieved with the purpose of guiding the data monitoring board on recommendations for the study. Missing data will be dealt with as missing covariate and outcome data. As a generalized estimating equations model will be used, missing outcome data will be dealt with using a missing at random assumption. If > 3% of data for covariate data is missing, multiple imputation will be used for the primary analysis. Given the way the adherence rate is computed, we anticipate that there may be a number of ways that the outcome measure could be missing. These include loss to follow-up and or partial information collected at anyone study visit. A further analysis will be performed in which missing primary outcome data will be imputed. Multiple imputation will be also performed on missing covariate and outcome data for all secondary clinical analyses in a similar way to that performed for the primary analysis.

## Monitoring

### Data monitoring: formal committee

The independent Data Monitoring Board (DMB) will make recommendations concerning the study to the Trial Steering Committee. The DMB is chaired by Dr. Simon Kasasa Senior Lecturer, in the Department of Epidemiology & Biostatistics at Makerere University School of Public Health in the College of Health Sciences, and other members include Dr. Suzanne Kiwanuka, a review Coordinator for Centre for Systematic Reviews on Human Resources for Health; Dr Paul Otuba, National MDR-TB Coordinator at the NTP; and Dr. Dathan Byonanebye, a lecturer in the Department of Community Health and Behavioural Sciences, Makerere University. The DMB is independent from the sponsor and has no competing interests.

### Data monitoring: Interim analysis

The first interim analysis will be conducted when 50% of the participants complete the study medication. Trial monitoring is conducted independent of the investigators by internal monitors at the Infectious Diseases Institute. The Trial Steering Committee is composed of Stavia Turyahabwe, program manager at the NTP in Uganda; Angom Esther, Nurse in charge at Lira Regional Referral Hospital; Dr Agnes Kiragga, a senior statistician with experience in HIV clinical trials and cohort studies; and Elizabeth Tindyebwa, a lay representative from the Friends’ Council (patient group) at the Infectious Disease Institute, Uganda. Since this trial is unblinded, the DMB will have access to intervention assignment. They will ensure that we protect the safety of the participants while assuring study integrity. The DMB will periodically (every 6 months) review the data, including interim analyses, and communicate any concerns to the study team and TSC.

### Harms

All observed or volunteered adverse events (AEs) regardless of treatment group or suspected causal relationship will be reported. During each study visit, the study doctors will assess AEs that may have occurred since the previous visit. The severity of adverse events will be graded according to the National Institute of Health Division of AIDS (DAIDS) classification system for reporting adverse experiences in adults [16].

### Auditing

An independent study monitor will be assigned to the study. The study monitor will perform at least two study audits annually to assess adherence to study protocol, SOPs, observe for study deviations and integrity of conduct of the study. The study monitor will provide a written report of their findings and recommendations after each study visit.

### Ethical considerations

The study has received approvals from the Mulago Hospital Research and Ethics Committee (Ref No: MHREC2125), the Uganda National Council for Science and Technology (Ref No: HS1796ES) and the National Drug Authority (RefNo:476/NDA/DPS/08/2021). The informed consent/assent document are in compliance with GCP and local regulatory authorities. Participants are taken through the consent process by the study team (Medical Officer or Study Nurse). Assent is requested from children between 8 and 17 years, and in addition, consent is sought from their parents or legally authorized representative. Informed consent is sought from participants aged 18 years and above and emancipated minors. Approval for protocol amendments shall be submitted to the Mulago Hospital Research and Ethics Committee for approval and then communicated to the National Drug Authority and The Uganda National Council for Science and Technology before implementation. Participants will be free to withdraw at any point of the study without any compromise to receiving their standard therapy. Children aged 8–17 years that do not provide assent but whose guardians have signed consent or vice versa will not be included in the study.

### Confidentiality

All study staff will ensure the protection of participant personal data and will not include participant names on any forms, reports, publications, or in any other disclosures.

## Declaration of interests

The authors have no competing interests to declare.

## Data access

Access to the database will be given to authorized personnel only (study investigators, project coordinator and data manager) and a log of authorized personnel will be stored in the trial master file.

## Dissemination plans

Dissemination workshops will be held with study participants, funders and other stakeholders to communicate the results of the study. The study results will be presented to officials in charge of the National TB Program in Uganda and at other national and international conferences. We shall also aim to publish in high-impact, peer-reviewed journals with a focus on open access. Full anonymity of participants’ details will be maintained throughout. We shall adhere to the International Committee of Medical Journal Editor (ICMJE) recommendations on the definition and roles of authorship. We shall not procure the services of professional writers.

## Availability of data and materials

Study data will be provided by the authors on request.

## Discussion

The introduction of new strategies like the use of shorter course regimen still does not address the operational limitations of DOT, including the unguaranteed accuracy of patient adherence information. In as much as MEMS technology may not be absolutely accurate in estimating patient adherence since opening the device may not imply drug ingestion, it does provide a patient-centred solution, relieving the burden on patients to meet an obligation of frequent clinic visits and maintaining patient autonomy [17]. MEMS technology also has the potential to meaningfully engage both caregivers and patients via its interactive functions such as LCDs on drug balances, reminders and real-time electronic display of patient adherence information. The adherence data abstracted from the MEMS technology enables caregivers to understand their patients’ adherence patterns and using this information, tailor counselling information, a better approach to improving adherence. The limitations of the MEMS technology include its inability to capture patient records on taking more than the required dosing and pill dumping [18]. The MEMS technology may also overestimate adherence by being unable to differentiate other instances in which the medicine devices are opened simply out of curiosity and altered behaviour as a result of being observed. The device may also be associated with inconvenience (on person carriage) and device fatigue which can negatively impact adherence [9]. Despite these limitations, this is one of the few studies that may address some of the facility and patient-based factors associated with non-adherence to MDR-TB therapy. It may also provide a cheaper and sustainable alternative for effective long-term follow-up of patients with MDR-TB with favourable outcomes including completion of therapy. The addition of pharmacokinetic data further strengthens adherence assessment in addition to the devices, patient self-reports and pill counts. We believe this study will demonstrate how MEMS technology may be used to remotely assess and therefore strengthen adherence in patients with tuberculosis for favourable treatment outcomes.

## Trial status

Protocol Version: 1.2 23.09.2021

MRHEC Ref No: MHREC2125

UNCST Ref No: HS1796ES

NDA Ref No: 476/NDA/DPS/08/2021

Start date: 15.11.2021

Approximate date for completion of recruitment: 31.01.2023

Participant enrolment is at 70% and expected completion is January of 2023.

## Supplementary Information

**Additional file 1.**

## Abbreviations

BDQ: Bedaquiline
CRF: Case Report Form
DAIDS: National Institutes of Health Division of AIDS
DMB: Data Monitoring Board
DOT: Directly observed therapy
DR-TB: Drug-resistant tuberculosis
DST: Drug susceptibility testing
EDP: Electronic DosePak®
GCP: Good Clinical Practice
GEE: Generalized estimating equations
HF-DOT: Health facility-based directly observed therapy
HIV: Human immunodeficiency virus
HPLC: High-performance liquid chromatography
IDI: Infectious Diseases Institute
LCD: Liquid crystal display
LFX: Levofloxacin
LZD: Linezolid
MDR-TB: Multi-drug-resistant tuberculosis
MEMS: Medications Events Monitoring System
MHREC: Mulago Hospital Research Ethics Committee
NTLP: National Tuberculosis and Leprosy Program
PK: Pharmacokinetics
PLHIV: People living with HIV
RR-TB: Rifampicin-resistant tuberculosis
SAE: Severe adverse event
SMS: Short Messaging Service
WHO: World Health Organization

## References

[1] Lange C, Dheda K, Chesov D, Mandalakas AM, Udwadia Z, Horsburgh CR (2019). Management of drug-resistant tuberculosis. The Lancet..

[2] Organisation WH. Global Tuberculosis Report. World Health Organisation; 2018.

[3] Uganda National TB and Leprosy Program Jul 2019 – June 2020 Report. Republic of Uganda Ministry of Health; 2020.

[4] Kizito E, Musaazi J, Mutesasira K, Twinomugisha F, Namwanje H, Kiyemba T (2021). Risk factors for mortality among patients diagnosed with multi-drug resistant tuberculosis in Uganda- a case-control study. BMC Infectious Diseases..

[5] Baluku JB, Nakazibwe B, Naloka J, Nabwana M, Mwanja S, Mulwana R (2021). Treatment outcomes of drug resistant tuberculosis patients with multiple poor prognostic indicators in Uganda: a countrywide 5-year retrospective study. J Clin Tuberc Other Mycobact Dis.

[6] Fiseha D, Demissie M (2015). Assessment of Directly Observed Therapy (DOT) following tuberculosis regimen change in Addis Ababa, Ethiopia: a qualitative study. BMC Infectious Diseases..

[7] Queiroz EM, De-La-Torre-Ugarte-Guanilo MC, Ferreira KR, Bertolozzi MR (2012). Tuberculosis: limitations and strengths of directly observed treatment short-course. Revista latino-americana de enfermagem..

[8] Pradipta IS, Houtsma D, van Boven JFM, Alffenaar JC, Hak E (2020). Interventions to improve medication adherence in tuberculosis patients: a systematic review of randomized controlled studies. NPJ Prim Care Respir Med.

[9] Shellmer DA, Zelikovsky N (2007). The challenges of using medication event monitoring technology with pediatric transplant patients. Pediatr Transplant..

[10] Schoenthaler A, Ogedegbe G (2008). Patients' perceptions of electronic monitoring devices affect medication adherence in hypertensive African Americans. Ann Pharmacother.

[11] Chan AW, Tetzlaff JM, Gøtzsche PC, Altman DG, Mann H, Berlin JA (2013). SPIRIT 2013 explanation and elaboration: guidance for protocols of clinical trials. BMJ (Clinical research ed)..

[12] Prasad R, Gupta N, Banka A (2017). Shorter & cheaper regimen to treat multidrug-resistant tuberculosis: a new hope. Indian J Med Res.

[13] WHO consolidated guidelines on drug-resistant tuberculosis treatment. Geneva; World Health Organisation; 2019. 30946559

[14] Vernon A, Fielding K, Savic R, Dodd L, Nahid P (2019). The importance of adherence in tuberculosis treatment clinical trials and its relevance in explanatory and pragmatic trials. PLoS Med.

[15] Nellums LB, Rustage K, Hargreaves S, Friedland JS (2018). Multidrug-resistant tuberculosis treatment adherence in migrants: a systematic review and meta-analysis. BMC medicine..

[16] Wandeler G, Mulenga L, Vinikoor MJ, Kovari H, Battegay M, Calmy A (2016). Liver fibrosis in treatment-naive HIV-infected and HIV/HBV co-infected patients: Zambia and Switzerland compared. Int J Infect Dis..

[17] Alipanah N, Jarlsberg L, Miller C, Linh NN, Falzon D, Jaramillo E (2018). Adherence interventions and outcomes of tuberculosis treatment: a systematic review and meta-analysis of trials and observational studies. PLoS medicine..

[18] Lyimo RA, van den Boogaard J, Msoka E, Hospers HJ, van der Ven A, Mushi D (2011). Measuring adherence to antiretroviral therapy in northern Tanzania: feasibility and acceptability of the Medication Event Monitoring System. BMC Public Health..

