# Supplementary material for: Comparing adherence to MDR-TB treatment among patients on self-administered therapy and those on directly observed therapy: non-inferiority randomized controlled trial
Source: Trials. 2023 May 12;24:326. doi: 10.1186/s13063-023-07314-z (PMC10176679; doi:10.1186/s13063-023-07314-z)
Supplement: Supplementary file 1 — Additional file 1. [file 13063_2023_7314_MOESM1_ESM.docx]

**Appendix A: - Informed Consent and Assent Forms**

**ADULT INFORMED CONSENT FORM**

**Title: Comparing adherence to MDR-TB treatment among patients on self-administered therapy and those on Directly Observed Therapy**

**Purpose:**

Tuberculosis is a lung infection which is very common in Uganda and spread through air droplets. When patients with TB skip some of their medication they may reduce the chances of the medication curing their TB infection. This condition is called drug resistant TB (MDR-TB). In addition, patients with drug- resistant infection can pass it on to others around them. MDR-TB requires longer duration of treatment and it is important for one to consistently take medication as required to ensure cure and prevent spreading infection to those around them. To ensure patients with MDR-TB adhere to their medication the Ministry of Health, Uganda adopted a system in which patients return to the health facility every day for a health care worker to observe them swallow medication for the entire duration of their treatment. This strategy is called Direct Observation Therapy (DOTS). This study is testing another method of ensuring that patients with MDR-TB can still daily take their medication even if they do not report to a health facility every day. We shall be using a technology like a sim card chip attached to the medication bottles and box of patients that automatically records the times they open their medication containers to swallow drugs. This is called MEMS technology. This method would imply that a patient can swallow their MDR-TB drugs at home and not require them to report every day to a health facility. They would however be required to report for scheduled monthly visits for drug refills and to have routine laboratory tests that inform the health care workers whether they are responding to their medication or not. We want to know whether remotely monitored patients (using MEMS technology) who self-administer MDR-TB drugs will have similar treatment completion rates compared to patients who report daily to health facilities to be observed as they swallow their MDR-TB drugs.

You have been selected because you tested positive for drug resistant TB and your doctor advises that you begin oral medication for the duration of 11-18 months. Please use every opportunity to get familiar with the study and ask any questions you may have.

**The estimated duration the research participant will take to in the research project:**

Should you decide to take part in the study, we shall collect information from you every single month for the duration of 11-18 months. Each study visit will take you approximately an hour.

**Procedures:** You can choose to take part in the study or not. It is your choice. If you choose not to take part in the study, there is no problem. If you choose to take part in the study, we shall enrol you from the time of initial admission in the ward for the first 2 months of treatment. Then after we shall ask you to attend the study clinic every month for the duration of your treatment.

**Visit 1 (screening visit):** On this day, we will determine if you are suitable to join this study. It is also the day that you will begin your treatment for MDR-TB. Those who are given the MEMS cap and box will be provided with information on how to use the MEMS devices (cap and box). They will be provided with medicine to last one month and asked to take the TB medicines in the morning between 6.30 am and 7.30 am every day.

Those who do not receive the MEMS cap and box will be given their medicine by the nurse or doctor every day between 6.30am and 7.30 am.

**Visit 2 and 3:**

- Visit 2 and 3 will be conducted after the first and second month of treatment and will take place in hospital. The following will take place;
- We shall ask you questions concerning your health.
- We shall provide you counselling to encourage you to swallow your medication everyday as prescribed
- Using your records at the TB clinic, we shall retrieve results of your sputum tests that are done every month by your doctor to monitor your response to treatment
- We shall provide you counselling to encourage you to swallow your medication everyday as prescribed.
- For those who have the MEMS devices, we shall retrieve information from your MEMS CAP device and box to understand how often you have been taking your medication and refresh your knowledge on the use of the MEMS devices (cap and box) We will also provide you a drug refill of MDR-TB drugs for the next month
- On visit 3, We shall collect a blood sample to measure MDR-TB drug levels in your blood

After 2 months of treatment, your doctor may decide to have you discharged to continue treatment from home.

***Visits 4-12:***

These visits will be at the end of each month (month 3 to month 11) of the treatment period. You will be expected to report to the study clinic (where you were admitted) on these visits. Those with the MEMS devices will be asked to carry their MEMS cap and box each time for the consequent scheduled visits for retrieval of data from the devices.

The following procedures will be conducted on these visits

- We shall ask you questions concerning your health.
- We shall provide you counselling to encourage you to swallow your medication everyday as prescribed.
- Using your records at the TB clinic, we shall retrieve results of your sputum tests that are done every month by your doctor to monitor your response to treatment
- We will also look at your medical records to help us understand your results better.
- For those who were given a MEMS cap and box will be asked about their experience using them and we shall retrieve information from your MEMS cap and box to understand how often you have been taking your medication. We shall pre-pack and clearly label your MDR-TB medication. The supply will be worth a month’s supply enough till your next clinic appointment
- On visits 9 and 12 corresponding to months 8 and 11 of treatment, we shall collect blood samples to measure MDR-TB drug levels in your blood.

***Blood draws at 2, 8 and 11 months of treatment (visits 3, 9 and 12)***

We shall only take blood samples from you on 3 of your 12 visits during the study. These will be at the end of months 2, 8 and 11 of treatment (corresponding to visit numbers 3, 9 and 12). The blood samples will be collected from you to measure the amount of MDR-TB drugs in your blood. Because of this, we shall ask that you do not swallow your MDR-TB drugs on the morning of these particular clinic visits but instead bring all medicine you take with you for your clinic visit. The blood samples collected on these visits by the study team will be separate from those required by your doctor.

Blood will be drawn from you (2) two times during each of these visits. The first blood sample will be collected just before you take your medicines. The study nurse will then give you your MDR-TB medicines to swallow and record the time you have taken the medication. Blood will then be collected 1 hour after you have taken your drugs. Approximately (2) two teaspoons of blood will be collected each time blood is collected from you meaning that we will take (4) four teaspoons of blood. Drinking water will be available and a light meal will be provided by the study at least one hour after you have taken your medicines. The study team will tell you when you can have a light meal.

**Risks/Discomforts:**

If you take part in this study, the risks to you are minimal. Most of the questions asked are general in nature. You are free to refuse to answer any questions. However, in order to have good results from the study, it is important that you attempt to answer all questions if possible. The entire research team has been trained to protect your privacy and all your information will be kept secret. As we collect blood samples from you we shall ensure this is done under very sterile conditions and by trained personnel. In the process you may however experience slight discomfort from the needle prick and minimal swelling at the site. You may experience stigma from other people when seen carrying medication containers. We shall provide you a tote bag in which to carry your medication containers.

**Benefits:**

Currently in Uganda there are no other systems in place that monitor adherence to MDR-TB treatment besides DOTS. The MEMS technology gives flexibility to the patient by allowing the doctor to monitor their adherence remotely. This study will help to provide information as to whether patients with MDR-TB can still adhere to their medication even when self-administering treatment just as well as those who report to a health facility daily for observation. The information you provide will help the Government of Uganda make a decision on the use of alternate methods for monitoring adherence to treatment among patients with MDR-TB by using methods that are cost effective to both the patients and the government and yet still provide favourable outcomes. By participating in this study, you will be monitored closely to determine whether or not your TB treatment is working.

**Confidentiality:**

Everything we talk about will be kept confidential to the extent allowed by the law. The information you provide will be kept confidential to the extent allowed by the law. To protect your identity, we will use a code number to identify you and all information. We will keep these records securely locked. Your name, or any other facts that might point to you, will not be evident or accessible when we present this study or publish its results.

**Alternatives:**

It is your decision as to whether to take part in the study or not. You can stop being in the study at any time you want without giving an explanation. This will not affect the standard medical services that you receive from your primary health care provider.

**Cost:** All testing will be conducted at no cost to you.

**Reimbursement:** For your study visit to the clinic we will reimburse you up to UGX 20,000/=

**Questions about the study:**

We would like to answer all your questions. If you have any questions now, please ask. If you have any questions in the future, you can contact:

• Dr Susan Adakun, Principal Investigator, TB Ward Mulago National Referral Hospital, Kampala Uganda. Telephone: 0772391455.

If you have any concerns about your rights in this study, please contact:

• Dr David Kateete, Chairperson of the IDI Research and Ethics Committee. Telephone: 0704879922

**Questions about participant’s rights:**

You have the right not to answer any questions you do not feel comfortable with. This decision will not affect the standard of care that you receive.

**Statement of voluntariness:**

Your participation in this study is voluntary and you are free to withdraw from the study at any time, without giving a reason and without your standard medical care or legal rights being affected.

**Ethical approval:**

This study will be conducted according to the principles of the Declaration of Helsinki for the study of human subjects. This study has received approval from the Infectious Diseases Institute Ethics Research Committee, Uganda National Council for Science and Technology (UNCST) and the National Drug Authority (NDA).

**Consent:** Statement of consent after understanding the study and a signature portion.

**STATEMENT OF CONSENT**

........................................................................... has described to me what is going to be done, the risks, the benefits involved and my rights regarding this study. I understand that my decision to participate in this study will not alter my usual medical care. In the use of this information, my identity will be concealed. I am aware that I may withdraw at any time. I understand that by signing this form, I do not waive any of my legal rights but merely indicate that I have been informed about the research study in which I am voluntarily agreeing to participate. A copy of this form will be provided to me.

Name ………………………Signature/thumb print of participant …………………Date ……

Name ………………………Signature of parent/guardian for minors (If applicable) …Date ……...

Name………………………Signature of witness (if applicable) ……………Date…………….

Name ………………………. Signature of interviewer/Person obtaining informed consent ……………………Date ………………….

**CHILD ASSENT FORM (ENGLISH)**

**AGES 8-17 YEARS**

**Study Title: Comparing adherence to MDR-TB treatment among patients on self-administered therapy and those on Directly Observed Therapy**

**1. These are some things we want you to know about this study:**

We are asking you to be in a research study. Research is a way to discover what people know think about a disease or do when they are sick of an illness. Research helps us learn new things and how to manage diseases in better way. It is your choice whether to be in this study. You can say Yes or No. Whatever you decide is OK. You will still receive the care you deserve.

Tuberculosis is a common lung infection that can be passed on from person to person. This disease causes someone to cough, have fevers in the evening, sweat a lot at night, lose appetite and lose weight. Healthcare workers know you have Tuberculosis if they find the germs that cause Tuberculosis in your sputum or if they examine you and find that you have some signs of TB like swollen lumps under your skin in the areas near your throat, under your arms or near your groin. When persons with Tuberculosis do not swallow their medication properly, their medication may stop working and they may develop tuberculosis that is resistant to common drugs. This is called drug-resistant Tuberculosis (MDR-TB). People with drug resistant TB require a different set of drugs to manage their infection and these are taken over an even longer period of time.

**2. Why are we asking you to be in this research study?**

You are being asked to participate in this research study because you tested positive for drug resistant TB and your doctor advises that you begin taking oral medication for the duration of 11-18 months.

**3. What is the study about?**

To make sure that patients with drug resistant TB swallow their medication as expected so that they can cure of their infection, they are required to report to a health facility everyday where a health worker watches them as they swallow their medication. This requires a patient to have resources to ensure they return to the health facility every day for the entire period of treatment. In this study we want to compare if patients who swallow medication on their own and use technology that can monitor whenever they take their medicine can complete their treatment just as well as patients who return to a health facility every day for supervised swallowing of medication. For the patients who swallow their medication on their own, we plan to attach a software device (MEMS technology) on the medicine containers that records and shows information on how many days a patient is swallowing their medicine.

**4. What will happen during this study?**

If you agree to be in this study,

At the start of the study;

1. If you are selected to use the MEMS technology, we will place a software chip on your medicine cap on your medicine bottle and another on the lid of your medicine box which will send us a signal whenever you open it to take your medicine.
2. If you are not selected to use the MEMS technology, you will report daily to the health facility near your home to receive your MDR-TB drugs as standard method of MDR-TB
3. We shall ask you to make one visit every month during the months of MDR-TB treatment
4. We shall ask you some information about your health
5. We shall instruct you on how to swallow your medication properly and give you information as to why that is important. At the end of each month of your treatment period;
6. We shall ask you questions on your health
7. We shall collect information from your TB treatment card to see how well you have been taking your MDR-TB medication in the last month
8. We shall instruct you on how to swallow your medication properly and give you information as to why that is important.
9. We will also look at your medical records at the TB clinic to help us understand your results better.

At the end of the 2^nd^, 8^th^ and 11^th^ months;

1. A day before these clinic visits we shall ask you not to swallow your MDR-TB medications on the morning of these visits, but request you to carry your drugs with you for your visit
2. We shall collect blood samples to measure the amount of MDR-TB drugs in your blood
3. The first blood sample will be collected just before you take your medicines. The study nurse will then give you your MDR-TB medicines to swallow and record the time you have taken the medication. Blood will then be collected 1 hour after you have taken your drugs.
4. We shall collect a total of 4 tsp spoons of blood each time

**5. Will it hurt you to be in the study?**

You may feel a little pain and or discomfort from the needle prick when blood samples are being collected. However, the people who will be drawing blood from you have been trained and will make sure it is done in a sterile way. Most of the questions asked are general in nature. You are free to refuse to answer any questions. However, in order to have good results from the study, it is important that you attempt to answer all questions if possible. The entire research team has been trained to protect your privacy and all your information will be kept secret. You may experience some stigma from other people who observe you carrying medicine containers. We shall provide you a tote bag in which you can privately carry your medication.

**6. What else should you know about the study?**

If you decide to participate in this study, you will make a total of 12 study visits, one each month over the course of your MDR-TB treatment. These visits are separate from your daily visit to the health facility from which you get your daily MDR-TB drugs. Your parent or guardian will not pay any money to the health workers for your participation in this study.

**7. What are the good things that might happen?**

People may have good things happen to them because they are in a study. These are called “benefits.” You shall have the benefit of learning more about the kind of TB you have from health workers and the how to take your medication correctly in order to increase your chances of curing it. The information you provide will help health workers and the Government of Uganda decide on other options of managing patients with drug resistant TB and still have good cure rates. Feel free to ask any question at the end of the interview.

**8. What if you don’t want to be in this study?**

You do not have to be in the study if you do not want to. Failure to be part of this study will not deny you access to health services that you have been receiving at your nearby health facilities You will still receive your medical care from your nearby health facility in the same way.

**10. Who should you ask if you have any questions about the study?**

If you have any questions about this study, you or your parents/guardians can call Dr. Susan Adakun Principal Investigator, TB Ward Mulago National Referral Hospital on 0772391455

And/or

Dr. David Kateete Chairperson of the IDI Research and Ethics Committee. Telephone: 0704879922

A copy of this assent form will be given to you.

**Statement of Child’s Assent**

Someone who works with the study has told me about the study. I have had a chance to ask questions and I know I can ask other questions at any time.

| ______________________________  Signature or thumbprint* of participant | *_________________________*  Printed name of participant | *___________________*  Date (day/month/year) |
| --- | --- | --- |
| ______________________________  Signature or thumbprint of parent or  guardian of the participant. | ______________________________  Printed name of the parent or guardian of the participant | ___________________  Date (day/month/year) |
| _______________________  Signature of person obtaining consent | *_________________________* Printed name of person obtaining consent | *__________________*  Date (day/month/year) |
| **If the parent or guardian of the participant is unable to read and/or write, an impartial witness should be present during the informed consent discussion.* | | |
| _______________________  Signature of witness | *_________________________* Printed name of Witness | *___________________*  Date (day/month/year) |
